# Failure Analysis for Overall Overturning of Concrete Single-Column Pier Bridges Induced by Temperature and Overloaded Vehicles

**DOI:** 10.3390/ma17112650

**Published:** 2024-05-30

**Authors:** Yelu Wang, Yongjun Zhou, Yuxin Xue, Changwei Yao, Kailong Wang, Xuchang Luo

**Affiliations:** 1 School of Highway, Chang’an University, Xi’an 710064, China; 2017021032@chd.edu.cn (Y.W.);; 2 School of Materials and Architectural Engineering, Guizhou Normal University, Guiyang 550025, China; 3 Jinan Urban Construction Group Co., Ltd., Jinan 250000, China; 4 Jinan Transportation Engineering Quality and Safety Center, Jinan 250001, China

**Keywords:** bridge engineering, single-column pier, collapse damage, overturning, temperature, mechanism

## Abstract

Several overloaded-induced overturning incidents of girder bridges with single-column piers have occurred in recent years, resulting in significant casualties and economic losses. Temperature, in addition to overloading, may also play a role in exacerbating bridge overturning. To investigate the association between temperature and bridge overturning, an explicit finite element model (EFEM) of a three-span concrete curved continuous bridge considering nonlinearities was developed to simulate overall collapse. The effects of uniform and gradient temperatures on the overall overturning stability of curved and straight bridges were evaluated based on the EFEMs. Furthermore, the temperature–bridge coupling model and temperature–vehicle–bridge coupling model were utilized to examine how gradient temperature influences bridge overturning. The results show that the overall overturning collapse of a bridge follows four stages: stabilization, transition, risk and overturning. Variations in uniform temperature from −30 °C to 60 °C had a negligible effect on the ultimate vehicle weight for bridge overturning, with a variation of less than 1%. As the gradient temperature ranged from −30 °C to 60 °C, curved bridges show less than a 2% variation in ultimate vehicle weights, compared to a range of −6.1% to 11.7% for straight bridges. The torsion caused by positive gradient temperature in curved bridges can exacerbate bridge overturning, while negative gradient temperature in straight bridges can lead the girder to ‘upward warping’, facilitating girder separation from bearings. Monitoring the girder rotation angle and vertical reaction force of bearings can serve as important indicators for comparing the stability of bridges.

## 1. Introduction

Bridges with single-column piers have been widely utilized in urban interchanges and highway engineering due to their lightweight, small occupation area, low cost, open space under the bridge, and strong adaptability [1,2]. When construction conditions are limited, bridges with single-column piers often emerge as the singularly viable solution [3].

Compared to double-column pier bridges, single-column pier bridges generally have poor anti-overturning capacity due to their single-support structural form in the transverse direction [4,5]. Over the past decade, several instances of an overloaded-induced overturning of the single-column pier bridge have occurred worldwide, particularly in China, resulting in significant casualties and economic losses [6,7,8,9]. Bridge overturning accidents are primarily caused by overloaded vehicles [5]. Previous studies primarily examined the ductility and strength of bridges with single-column piers, while neglecting their lateral stability. These incidents have prompted bridge engineers to reconsider the design of single-column pier structures, enhancing the original theory and emphasizing the importance of lateral overturning stability [10].

Due to the inherent risk of overturning in this type of bridge, numerous researchers have utilized theoretical analysis, field investigations, experiments, and finite element techniques to explore the associated overturning mechanisms and failure modes [11,12,13,14,15]. Field investigations have revealed four common characteristics of bridges involved in overturning incidents [16,17,18]: (1) continuous girder bridges, generally with a single bearing at each mid-pier; (2) superstructures made of integral box-section girders; (3) straight girders or horizontally curved girders with a large curvature radius; and (4) overloaded vehicles moving or staying on the same deck side. The anti-overturning theory of the single-column pier bridges has evolved from rigid body rotation theory to deformed body and rigid body rotation superposition theory [9,19,20]. Xiong [21] established a detailed 3D bridge model including the superstructure, bearings, and piers to accurately describe the structural behavior under different mechanical conditions before the final collapse, and further explored the effect of different single-column pier bridge arrangements on overturning performance. Peng [10] established nonlinear 3D FE models of the collapsed Chunhui and Hongfu bridges to verify the accuracy of the proposed AOLA method in conjunction with the data and photographs collected from the field investigation. Shi [22] used an explicit nonlinear dynamic finite element model (EFEM) to analyze the incidents of highway ramp bridges and revealed the causes of bridge overturning by simulating the collapse process.

FE models that consider geometric nonlinearity, material nonlinearity, and dynamic effects are complex and computationally intensive [2,20]. Therefore, researchers adopt the support reaction method (SRM) or the factor of stability against overturning (FSO) [23] to address the overturning issues in practice. Some studies have proposed that the overturning of single-column pier bridges can be assessed based on the presence or absence of a disengaged bearing, indicating whether there is a negative support reaction [5,20]. While preventing the disengagement of all bearings in the limit state may avoid bridge overturning incidents, this approach does not provide a quantitative measure of the bridge’s anti-overturning ability under overloaded vehicles. FSO is defined as the ratio of the anti-overturning moment caused by dead loads to the overturning moment caused by vehicles. A girder will overturn if the FSO is below one [9]. The key aspect of the FSO lies in determining the critical state of overturning and selecting the position of the overturning axis [15,24]. However, researchers discovered that the FSO of several overturned multi-span bridges exceeds one when based on a rigid overturning axis, indicating its unsuitability for multi-span bridges [9]. Li [25] proposed a modified overturning axis for the FSO considering the effects of deformed body rotation and rigid body rotation. The updated FSO method takes the line of the outermost bearing of each support section as the overturning axis (fold overturning axis), which has been integrated into the MTPRC2018 code [26]. Zhou [3] and Liu [20] highlighted that while the updated FSO method appears reasonable, it still presents several limitations. The method solely focuses on the role of torsion-resisting bearings and ignores the contribution of non-torsion-resisting bearings in anti-overturning during girder rotation. Furthermore, the arrangement of vehicles is based on the most unfavorable state of the reaction force, which may not align with the actual most critical position. Additionally, the method fails to account for the alteration in shape and volume of the bearing as the superstructure undergoes overturning [19,21]. These apparent drawbacks may cause total disagreement between the safety assessment results obtained according to specifications and the process of overturning.

In addition to bridges being threatened by overloaded vehicles, temperature has also garnered significant attention for its detrimental effects [27,28,29]. Bridges exposed to the atmosphere are subject to thermal energy exchange between their surfaces and the surrounding environment, resulting in time-varying thermal loads on the bridge [30]. He [31] reported an incident in which the bridge experienced lateral creep due to temperature, eventually causing the bridge to overturn. The girder will move outward along the radial direction under the coupling effects of vehicle and temperature, and the phenomenon of ‘crawling’ will occur [12]. To date, the effect and mechanism of temperature on the overturning stability of single-column pier bridges are still controversial. Qi [32] calculated the stability of the two bridges using the FSO; the temperature-induced overturning effect for straight steel box girder bridges varied from 3.3% to 6.9% of the vehicle, while that for curved steel bridges was 109.1% to 356.3%. Li [33] analyzed the effect of gradient temperature on the overturning stability of bridges using a rigid overturning axis. The results showed that the gradient temperature only caused bearing disengagement and bearing reaction force redistribution, without any influence on the FSO. In contrast, other scholars [34,35] have concluded that temperature-induced variations in bearing reactions ultimately lead to a reduction in the overturning moment of the bridge, and thus temperature is a disadvantage for bridge stability. Wang [27] conducted an anti-overturning analysis combined with temperature field measurements on a three-span curved steel box girder bridge, where separating displacements due to temperature variations were predicted. For continuous girder bridges, according to the static equilibrium theory, the sum of the vertical bearing reaction force induced by temperature is zero, which theoretically causes only a bearing reaction force redistribution without torsional moments generated on the overturning axis. However, a greater temperature-induced overturning effect is obtained by utilizing the updated FSO method, and oversimplified computational methods and models are the underlying cause for the different conclusions.

There is no consensus regarding the overturning mechanism of temperature on the bridges. Compared with linear models or methods, conclusions obtained from nonlinear-based bridge overturning collapse inversion techniques have higher credibility. Although some progress has been made in the field of temperature-induced bridge overturning, further studies using refined finite element techniques are still needed to investigate the relationship between temperature and bridge overturning due to the employment of linear models and simplified computational methods. The main contributions of this study are as follows: (1) the bridges were systematically classified based on the location of the last failed bearing; (2) the EFEM was used for the first time to explore the effect of uniform temperature and gradient temperature on the overall overturning stability of bridges; and (3) it was found that torsional deformation in curved bridges and vertical deformation in straight bridges are the primary reasons temperature affects bridge overturning stability. The outline of the paper is as follows: Section 2 provides a detailed analysis of the process of the overall overturning of a three-span curved concrete continuous box-girder bridge using ANSYS/LS-DYNA 2023 R1 software. The response and failure characteristics of the bridge are described. In Section 3, the EFEM of curved and straight concrete continuous bridges were developed, contact and geometric nonlinearities were considered, and the effects of two typical temperature modes (uniform and gradient temperature) on the overall overturning stability of the bridges were investigated. Section 4 used the temperature–bridge coupling model and the temperature–vehicle–bridge model to elucidate the overturning mechanism of the bridge induced by gradient temperatures from the perspectives of girder deformation, rotation angle, and bearing reaction forces. Finally, Section 5 concludes the study.

## 2. Numerical Analysis of the Overturning Process

EFEM is often used for simulating structural collapse processes, allowing for the capture of transient states during collapse [36]. EFEM primarily utilizes the central difference method, which offers higher computational efficiency compared to implicit dynamic algorithms and is well-suited for simulating the discontinuous nonlinear behavior of structures. EFEM has been successfully implemented in many cases. Shi [22] used EFEM to analyze the entire overturning and collapse process of the Yuegan Expressway ramp bridge. Ji [12] carries out a full-range nonlinear analysis of the lateral overturning process of a three-span curved steel–concrete composite box girder based on the EFEM. Liu [20] analyzed the entire overturning process of an example bridge based on EFEM and determined the validity of the overturning risk assessment method with the comparison of the evaluation results under critical loads. All of the above analyses demonstrate the reliability of EFEM in simulating the bridge overturning process. In this section, an explicit dynamic finite element analysis of a three-span curved concrete box girder bridge is carried out using ANSYS/LS-DYNA software; the model size, material parameters, boundary conditions, and other related information are described.

### 2.1. Prototype Bridge

**Figure 1:**
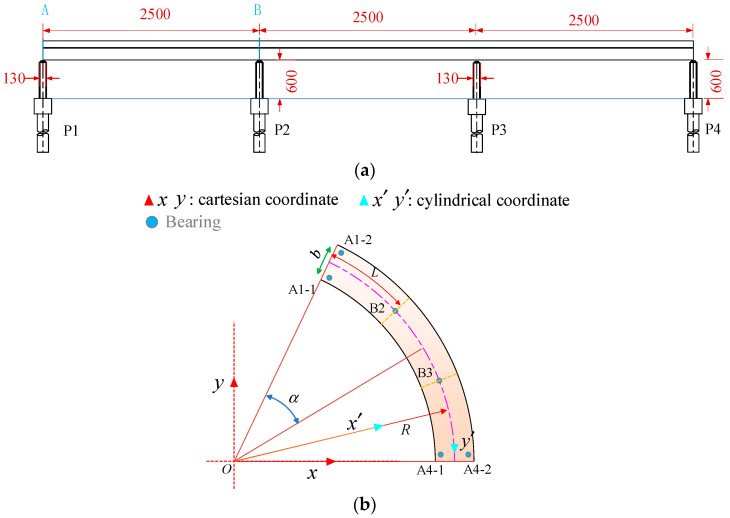
Layout of the modeled bridge (unit: cm): (**a**) elevational dimensions and (**b**) plan dimensions. Notes: A and B represent section IDs, respectively.

**Figure 2:**
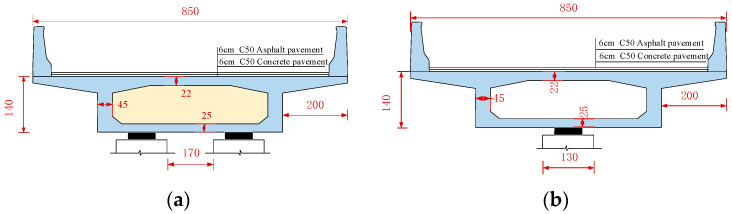
Girder cross-section (unit: cm): (**a**) Section A and (**b**) Section B.

A 3 × 25 m prestressed concrete single-column pier girder bridge on an expressway interchange ramp was selected as an example. Figure 1 presents the layout and support form of the bridge, which has a radius of curvature of 80 m and a central angle of 53.7° for the girder. The bridge has four piers, with a height of 6 m and a diameter of 1.3 m. Piles are constructed beneath the piers. Among them, piers P2 and P3 are single-column piers, each with a bearing B2 or B3, while piers P1 and P4 are double-column piers with two plate rubber bearings A1-1 and A1-2, or A4-1 and A4-2, installed at a distance of 2.8 m apart. For bearings, A represents the side-pier bearing while B represents the mid-pier bearing. The diameter and thickness of each bearing are 60 cm and 19 cm, respectively. Piers and bearings are constructed perpendicular to the centerline of the road. The superstructure girder is a single box with one chamber, maintaining a height of 1.4 m along the curve direction. The concrete top, bottom, and web slabs have thicknesses of 22 cm, 25 cm, and 45 cm, respectively. The detailed cross-sectional dimensions of the girder can be seen in Figure 2.

### 2.2. Material Parameters

Many on-site investigations suggest that the structural damage from overturning is primarily due to overall instability. As a result, there has been a heightened focus on the stability of structures. Therefore, the superstructure can be simplified to a homogeneous elastic material model. The girder was constructed of C50 concrete with a density of 2650 kg/m^3^, an elastic modulus of 34.5 GPa, and a Poisson’s ratio of 0.2. The piers were composed of C40 concrete with a density of 2650 kg/m^3^, an elastic modulus of 32.5 GPa, and a Poisson’s ratio of 0.2. The anti-collision guardrail was also made of C50 concrete with an elastic modulus of 34.5 GPa. Additionally, the bearing had an elastic modulus of 2.0 GPa, a density of 1400 kg/m^3^, and a Poisson’s ratio of 0.49. The truck’s tires were constructed from rubber material modeled with elastic properties. The tires had a density of 1400 kg/m^3^, a modulus of elasticity of 11.0 GPa, and a Poisson’s ratio of 0.3. Similarly, the truck body was also simulated using an elastic material. Since a vehicle with a normal weight is not suitable for anti-overturning analysis, its density was determined by the ultimate weight and volume of the truck. As the material properties of the truck body have little influence on the mechanical behavior of the bridge, an elastic modulus of 11.0 GPa and a Poisson’s ratio of 0.3 were selected for the truck body.

ANSYS/LS-DYNA software was adopted to model the bridge. In the FE model, a SOLID164 element defined by eight nodes was utilized to simulate the girder, piers, bearings, wheels, and truck body. The mesh size for the bearings was set to 0.03 m, while that for the girder, piers, anti-collision guardrails, and the truck body was 0.5 m. To avoid larger hourglass effects, the mesh size for the bearings and wheels was set to 0.1 m. The bridge model consisted of 114,726 elements, while the truck model had 17,046 elements. Both the bridge and truck components were simulated based on their actual dimensions, with material properties specified and components assembled accordingly.

**Figure 3:**
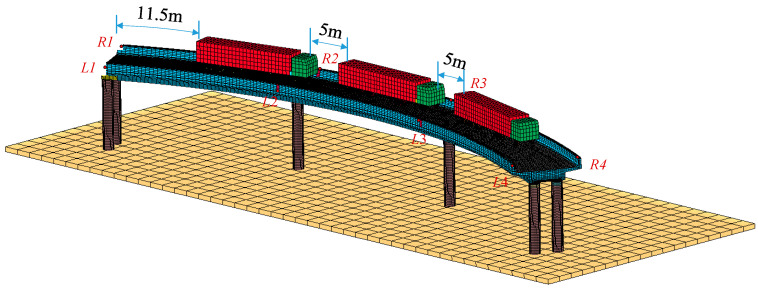
Refined FE model of the 3 × 25 m curved single-column pier bridges.

It was assumed that the bottoms of all piers were completely fixed. Surface-to-surface contact was utilized to simulate the contact characteristics between piers and bearings, bearings and girders, and wheels and concrete slabs. To account for potential collisions between bridge parts during the overturning process, the entire model was set to erosion contact in the event of mutual penetration between parts. The contact keyword for the trucks and girder was *CONTACT_AUTOMATIC_SURFACE_TO_SURFACE_ID, while the contact keyword for the girder and bearings, and bearings and piers was *CONTACT_ERODING_SURFACE_TO_SURFACE_ID. The maintenance of contact can be determined by whether the contact force is zero, allowing for the identification of bearing disengagement. The tangential contact relationship is modeled using a penalty function. Figure 3 illustrates the coupled FE model of the truck and bridge.

### 2.3. Load Setting

The load of the model consisted of two components: the structure’s self-weight and the truckload. The self-weight was obtained by applying the gravity acceleration of 9.8 m/s^2^ in the model. Three six-axle trucks were arranged in dense rows for loading vehicles. The weight distribution ratios for the first six axles were 0.04, 0.19, 0.17, 0.21, 0.19, and 0.21. The total length of the vehicle was 15 m with axle spacings of 3.4 m, 0.9 m, 4.6 m, 1.6 m, and 1.6 m. Vehicle locations were determined by the most unfavorable influence surface of the total overturning effect of the bridge [20], which is shown in Figure 3. The distance between the front and rear vehicles was set at 5 m, with the vehicle positioned 0.5 m from the curb. To ensure accurate calculations and avoid convergence issues and structural vibrations, gravity was gradually applied to the structure from 0 s to 1 s. The quasi-static effect of the bridge involves implementing critical damping, where the damping coefficient is set at twice the angular frequency of the bridge. When performing simulation, the model is controlled by the *CONTROL_HOURGLASS keyword with rigid hourglass control enabled and a coefficient of 0.04. Mass scaling was achieved using the *CONTROL_TIMESTEP keyword set to a negative value, ensuring that the ratio of added mass to energy in the model was kept within 5%.

### 2.4. Overturning Process and Failure Characteristics

**Figure 4:**
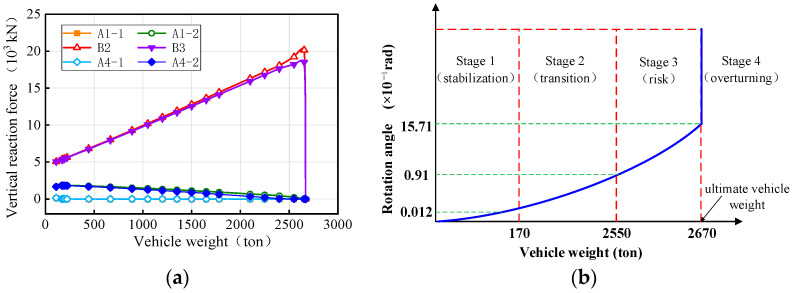
Overturning effects of the bridge under different trucks: (**a**) vertical reaction force of the bearings and (**b**) rotation angle of the girder.

A massively parallel processing (MPP) distributed model was utilized to conduct overturning analysis on workstations equipped with 64 cores and 128 threads. During the process of the vehicle crushing the bridge, the weights of the trucks were continuously adjusted to observe changes in the vertical reaction force of the bearings and the rotation angle of the girder. These observations were recorded and plotted in Figure 4. Upon reaching a certain truck weight, the weight of the truck when the bridge initially overturns was referred to as the vehicle ultimate weight. The condition of the bridge when subjected to the vehicles with this ultimate weight was then denoted as the overturning limit state. Figure 4a shows that as the vehicle weights increase, the vertical reaction force of the mid-pier bearings (B2 and B3) continues increasing, whereas the vertical reaction force of the side-pier bearings gradually decreases. Bearings A1-1 and A4-1, located on the inner horizontal curvature of the girder, detached first, followed by bearings A1-2 and A4-1. Subsequently, when bearings B2 and B3 fail, the bridge transitions into a two-point support system, resulting in bridge overturning collapse.

By summarizing, as shown in Figure 4, the overturning damage process of bridges can be divided into four stages: stabilization, transition, risk, and overturning. During the stabilization stage, the bridge bearings are fully effective under the vehicles, and the girder has minimal deformation. The stage ends when one of the bearings fails, corresponding to a girder rotation angle of 0.0012 rad. Subsequently, as the trucks’ weights increase, multiple bearings fail, marking the transition stage. In this stage, the number of effective bridge bearings remaining is three or more and the girder remains stable. At the end of this stage, the bridge boundary conditions degrade to a three-bearing support system, and the girder rotates by 0.09 rad. Further, by increasing the weights of the vehicles, the bridge enters the risk stage, where the number of effective bearings remains at three and the girder faces a heightened risk of overturning. Finally, when the bridge is supported by two bearings, the overturning effect exceeds the anti-overturning effect, leading to divergent displacement and an overall girder overturning, which is denoted by the overturning stage. Consequently, the state corresponding to the two bearing support forms of the bridge can be used as a failure criterion to determine the limit state of the bridge’s overall overturning. According to the Chinese Bearing Design Code (JT/T 391-2009) [37], the girder (bearing) is designed to have a rotation angle within 0.02 rad. In conjunction with Figure 4b, it can be seen that this measure can prevent the bridge from entering the risk stage.

**Figure 5:**
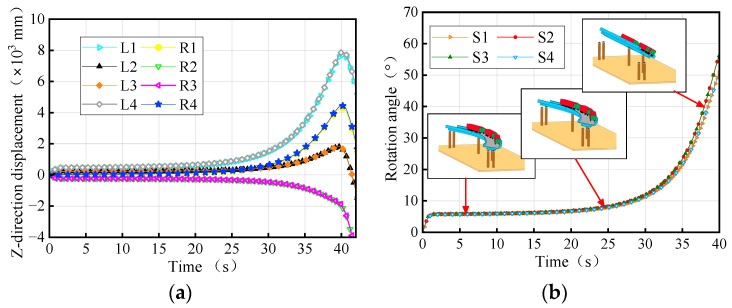
Overturning effects of the 3 × 25 m curved bridge: (**a**) z-direction displacement of the girder and (**b**) rotation angle of the girder. Note: $L_{i}$ and $R_{i}$ represent the left and right sites of the girder section above the ith pier position, respectively, and $S_{i}$ represents the girder section above the ith pier.

**Figure 6:**
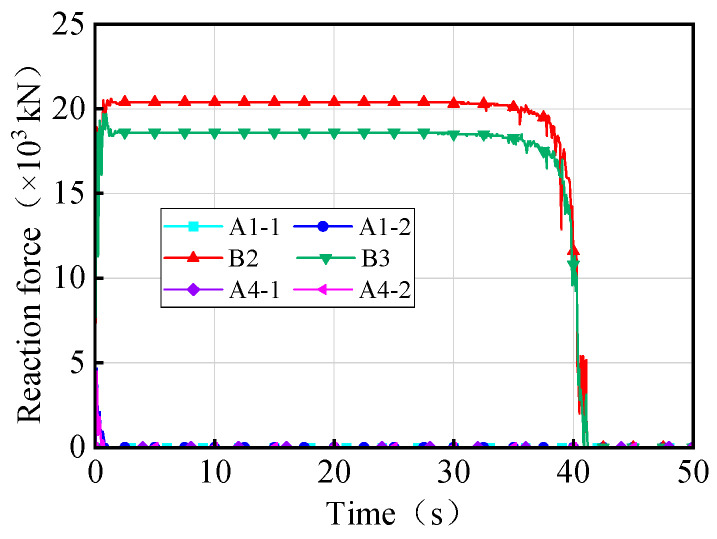
Bearing reactions of the 3 × 25 m curved bridge subjected to trucks.

**Figure 7:**
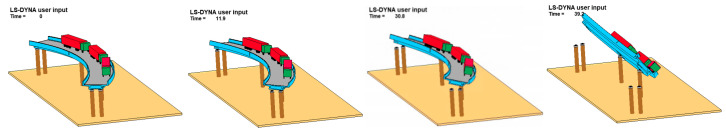
Overturning process of the 3 × 25 m single-column pier bridges.

Figure 5 presents the time history of the displacement and rotation angle of the 3 × 25 m curved bridge subject to three trucks with ultimate weights (overturning limit state). The rotation angles of different girder sections exhibit similar trends and magnitudes, with a maximum difference of 6°. This conclusion is consistent with reference [9]. The angle of rotation of the girder follows an ‘S’ shape pattern during overturning, as shown in Figure 5b. Additionally, in Figure 6, individual bearings of the bridge gradually fail under truck loading. The failed bearings start from the inner curvature or non-overturning side and progress towards the overturning side, leading to evolving support conditions. As the bridge support condition deteriorates to two bearings, the girder rotation angle increases rapidly, displacements diverge, and the bridge experiences overall overturning damage. The overall overturning process of the bridge can be illustrated in Figure 7.

## 3. Overturning Effects of Bridge in Temperature Field

Further, numerical methods were used to analyze the overall overturning process and the influence of straight and curved bridges under the coupling effect of temperature and overloaded vehicles, with a focus on the overturning limit state.

### 3.1. Temperature Model

The effect of temperature on bridges typically involves two aspects: gradient temperature and uniform temperature [38]. Gradient temperature, influenced by sunlight variations, can lead to the warping or torsion of the girder, while uniform temperature changes due to seasonal temperature variations can result in a plane deformation of the girder. For gradient temperatures, early scholars assumed that the temperature distribution was linear along the depth of the concrete box girder, but subsequent experimental studies revealed a nonlinear distribution that varies both along the girder depth and over time [39]. Understanding the temperature field within a concrete structure is crucial for calculating temperature loads [40]. Assuming that the temperature T at any given point inside or on the surface of a concrete member at a specific moment can be mathematically expressed as follows:

(1) T=f(x,y,z,t)

The temperature T at a certain point is determined not only by its coordinates x, y, and z, but is also related to time t. According to the heat conduction theory, a transient thermal conductivity equation can be derived for homogeneous, isotropic concrete through elastodynamic principles [27]:

(2) $$\lambda \left(\frac{\partial^{2}T}{\partial x^{2}}+\frac{\partial^{2}T}{\partial y^{2}}+\frac{\partial^{2}T}{\partial z^{2}}\right)=c\gamma \frac{\partial T}{\partial t}-q$$

where λ is the concrete thermal conductivity; c is the specific heat of concrete; γ is the bulk density of concrete; and q is the heat generation per unit volume of concrete.

When the hydration heat of the concrete structure is not considered, the temperature field coordinate function at the initial moment of heat conduction is as follows:

(3) $$\lambda \left(\frac{\partial^{2}T}{\partial x^{2}}+\frac{\partial^{2}T}{\partial y^{2}}+\frac{\partial^{2}T}{\partial z^{2}}\right)=c\gamma \frac{\partial T}{\partial t}$$

(4) $$T(x,y,z,t=0)=T_{0}(x,y,z)$$

When the girder height is small, heat conduction in the vertical direction outweighs that in the horizontal direction. Therefore, the horizontal heat conduction can often be disregarded, allowing for an approximate analysis based on one-dimensional heat conduction in the vertical direction. In the case of higher girders, neglecting the complex heat conduction near the corner zone enables calculations to be approximated by the superposition of one-dimensional heat conduction states in both vertical and horizontal directions. This simplifies the temperature field calculation to a one-dimensional heat conduction problem:

(5) $$\alpha \frac{\partial^{2}T}{\partial x^{2}}=\frac{\partial T}{\partial t}$$

where α is thermal conductivity, α=λ/cγ.

Assuming that concrete is a semi-infinite thick slab and the temperature variation is in a harmonic form, the elastic mechanical solution of the thermodynamic issue with the first type of boundary condition can be obtained using Equation (5) [41]:

(6) $$T(x,t)=A_{0}e^{-x\sqrt{\omega /2\alpha }}\sin(\omega t-x\sqrt{\omega /2\alpha }x)$$

where $A_{0}$ is the peak temperature fluctuation on the slab surface; ω is circular frequency, ω=2π/24; and x represents distance from the calculation point to the surface of the slab.

For engineering applications, the maximum temperature distribution effect at a certain moment is typically used as the design load, allowing Equation (6) to be represented in the form of a temperature distribution envelope:

(7) $$T(x)=A_{0}e^{-\sqrt{\omega /2\alpha x}}$$

Liu [42] developed the nonlinear pattern by modeling box girders on viaducts and employed the following equation to calculate the temperature distribution along the slab thickness:

(8) $$T(x)=T_{0}e^{-c_{z}x}$$

where $T_{0}$ is the temperature difference between the inner and outer surfaces of the member, and $c_{x}\approx 10$.

**Figure 8:**
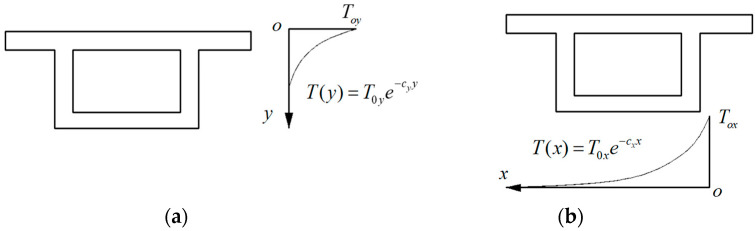
Distribution models of gradient temperatures: (**a**) temperature distribution along girder height and (**b**) temperature distribution along horizontal.

The analysis of existing measured data reveals that the temperature difference distribution pattern along the direction of box girder height and girder width closely resembles Figure 8. This pattern can be calculated using Equations (9) and (10):

(9) $$T(y)=T_{0y}e^{-c_{y}y}\;$$

(10) $$T(x)=T_{0x}e^{-c_{x}x}$$

where $T_{0y}$ is the temperature difference between the top and bottom of the box girder; $T_{0x}$ is the temperature difference between the two outer webs of the box girder; $c_{x}$ and $c_{y}$ are exponential coefficients, which are related to the structural form, calculation position, and time; and x and y are the distances from the calculation point to the heated surface.

**Figure 9:**
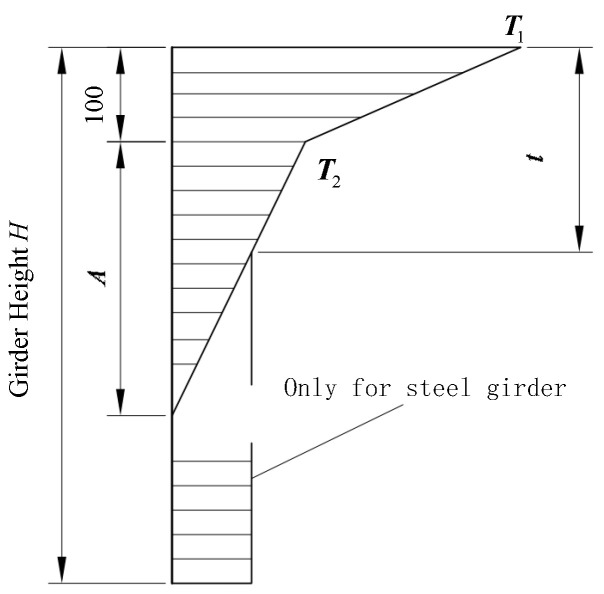
Temperature distribution in MTPRC2015 code (unit: mm).

Presently, many bridge design codes simplify the complex temperature field to a one-dimensional problem [30]. The British BS5400 specification is considered the most comprehensive guideline for determining temperature loads on bridge structures, as it considers daily and seasonal variations in temperature, solar radiation, and reverse radiation. The temperature difference distribution between ascending and descending temperatures specified in the MTPRC2015 code [43] is confined to the upper slab portion of the box girder, which follows a rectangular ±5 °C distribution, depicted in Figure 9. Because of the many overturning incidents that occurred in China, the temperature models outlined in the MTPRC2015 code were utilized for subsequent numerical simulations.

### 3.2. Uniform Temperature Effects

The finite element method is an effective method to analyze the temperature distribution of some important structures. Assuming an initial temperature of zero for the bridge, the girder gradually heats up and cools down as a whole within a time frame of 0 s to 1 s, taking into account the effect of gravity on the structure. Through extensive research on multi-country standards, extreme temperature conditions of heating 20 °C, 40 °C, and 60 °C, as well as cooling −10 °C, −20 °C, and −30 °C, were considered. In this study, uniform temperature action was applied to all node groups of the superstructure girder in the model to simulate steady-state thermal effects. The keyword of the thermodynamic model in ANSYS/LS-DYNA is *MAT_ELASTIC_PLASTIC_THERMAL. Other modeling details are consistent with Section 2.1, Section 2.2, Section 2.3, Section 2.4.

**Figure 10:**
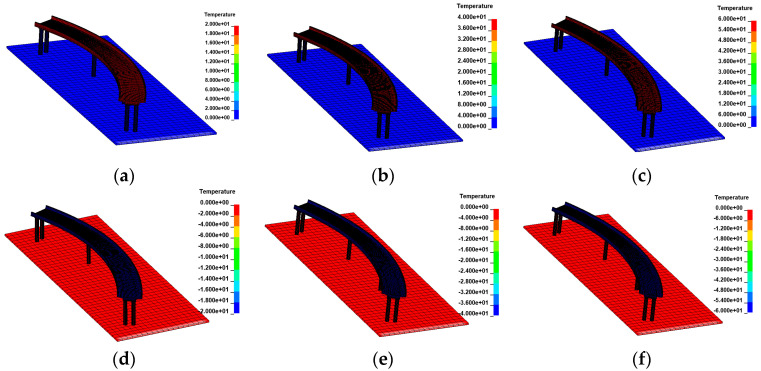
Thermodynamic FE models of the 3 × 25 m curved bridge subjected to uniform temperatures: (**a**) heating 20 °C; (**b**) heating 40 °C; (**c**) heating 60 °C; (**d**) cooling 10 °C; (**e**) cooling 20 °C; and (**f**) cooling 30 °C.

Previous studies have demonstrated that straight and curved bridges exhibit distinct overturning characteristics [18,22,44,45]. In straight bridges, the side-pier bearing is the last to fail in the overall overturning process, whereas in curved bridges, it is the mid-pier bearing. The critical radius of horizontal curvature is defined as the radius at which the connection line between the outermost bearings of each bridge pier becomes straight. This critical radius of horizontal curvature serves as a criterion to differentiate between straight bridges and curved bridges; bridges with a radius of curvature greater than the critical value are classified as straight bridges, while those with a radius of curvature less than the critical value are considered curved bridges. In practice, the horizontal curvature radius of the bridge is typically set at 80 m or more to accommodate the turning of the trailer. Based on the above reasons, curved bridges with a curvature radius of 80 m and straight bridges with a curvature radius of 10,000 m were selected to investigate the effects of temperature and overturning caused by overloaded vehicles. Additionally, three-span and five-span bridges with 25 m span lengths were included for a more comprehensive analysis. The thermodynamic FE model of a 3 × 25 m bridge subjected to uniform temperature is shown in Figure 10.

**Figure 11:**
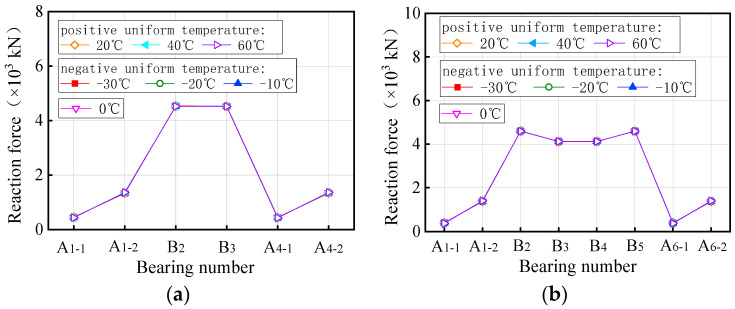
Vertical reaction force of the curved bridges subjected to uniform temperatures: (**a**) 3 × 25 m bridges and (**b**) 5 × 25 m bridges.

Figure 11 presents the vertical bearing reaction force of the curved bridges under self-weight and uniform temperature. Under positive uniform temperature conditions, the vertical reaction force of bearings A1-1 and A4-1 of the 3 × 25 m curved bridge gradually decreases with the overall increase in temperature, showing a decrease of 2.2% at a uniform temperature condition of heating 60 °C. Conversely, the vertical reaction force of bearing A1-2 shows an opposite trend to that of bearing A1-1, increasing by 1.3% at the same heating condition. Bearings B2 and B3 exhibit a similar variation pattern to A1-1, with a decrease of 0.1% at the limit condition of heating 60 °C. For a 5 × 25 m curved bridge, the vertical reaction force of the bearings (A1-1, A6-1) on the outside of the curve decreases gradually with rising temperature, while the vertical reaction force of the bearings (A1-2, A6-2) on the inside of the curvature gradually increase. The vertical reaction force of the bearings B2 and B5 on the mid-pier gradually decreases with increasing temperature, while those of the bearings B3 and B4 increase. The vertical reaction force of all bearings for the 5 × 25 m curved bridge, under a 60 °C heating condition, varies from −6.1% to 2%. Furthermore, the vertical reaction force of bearings for both the 3 × 25 m and 5 × 25 m curved bridges, under negative uniform temperature, exhibit an opposite pattern compared to positive uniform temperature conditions. Specifically, under a cooling condition of 30 °C, the vertical reaction force of all bearings for the two curved bridges varies from −1.2% to 2.3% and −0.5% to 1.8%, respectively.

**Figure 12:**
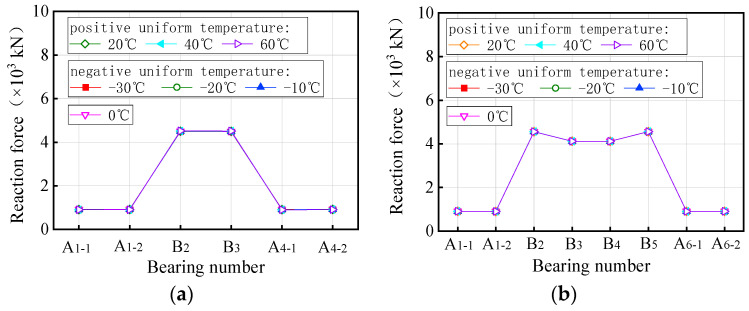
Vertical reaction force of the straight bridges subjected to uniform temperatures: (**a**) 3 × 25 m bridge and (**b**) 5 × 25 m bridge.

Figure 12 illustrates the vertical bearing reaction force for straight bridges. The vertical reaction force of bearings A1-1, A1-2, A4-1, and A4-2 on the 3 × 25 m straight bridge decreases gradually under positive uniform temperature conditions, with a maximum variation of 1.1% at a uniform temperature condition of heating 60 °C. Conversely, the vertical reaction force of bearings B2 and P3 shows an opposite trend, increasing by up to 0.4% at the same heating condition. Under negative uniform temperature, the vertical reaction force of the bearings exhibits a reverse variation pattern compared to the positive uniform temperature condition. Vertical reaction forces for all bearings of the 3 × 25 m straight bridge varied from −6.1% to 2% at temperatures ranging from −30 °C to 60 °C. Similarly, for the 5 × 25 m straight bridge, under a temperature range of −30 °C to 60 °C, the vertical reaction force varies from −6.1% to 2%. Further analysis reveals that the total vertical reaction force of all bearings remains constant and unaffected by temperature changes. In conclusion, uniform temperature only leads to a redistribution of vertical bearing reaction force in both straight and curved bridges, without introducing additional force to the structural system.

**Figure 13:**
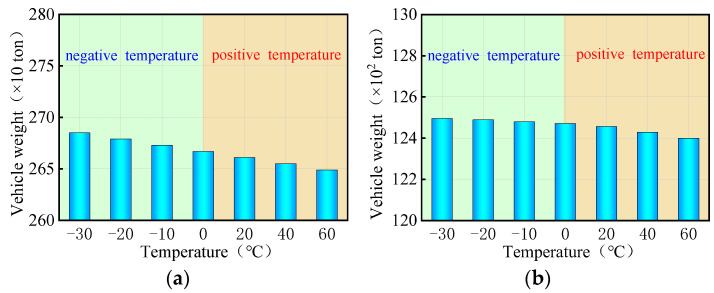
Ultimate vehicle weights of the overall overturning of the curved bridges subjected to trucks and uniform temperatures: (**a**) 3 × 25 m bridge and (**b**) 5 × 25 m bridge.

Currently, there are two indicators to evaluate the overall anti-overturning stability of a bridge: the stability coefficient and the ultimate vehicle weight. The stability coefficient is based on a simplified anti-overturning calculation method, which is unreliable due to disclosed flaws. On the other hand, a refined FE model provides a more direct reflection of the actual anti-overturning performance of the bridge as it takes into account the interaction between the vehicles with the ultimate weights and the bridge. In the study involving a coupled model of bridge and temperature, three trucks with six axles were utilized, with their weights adjusted by varying the density of the truck body until the bridge overturned. The overall anti-overturning performance of a bridge was assessed by the ultimate vehicle weights obtained from iterative calculations. Figure 13 presents the ultimate vehicle weights of the curved bridges subjected to three trucks and uniform temperatures. Figure 13 shows the limiting vehicle weights for a curved bridge subjected to three trucks and uniform temperatures. The variations in vehicle weights in Figure 13 were achieved by adjusting the truck body density. For the curved bridges measuring 3 × 25 m and 5 × 25 m, the ultimate vehicle weights decrease with increasing positive uniform temperatures, showing a decrease of 0.7% at the ultimate uniform temperature of 60 °C. Conversely, under negative uniform temperature conditions, the overall overturning ultimate vehicle weights of the two bridges gradually increases as the temperature decreases. Within the temperature range of −10 °C to −30 °C, the ultimate vehicle weights of the two curved bridges increase by 0.1% to 0.7%.

**Figure 14:**
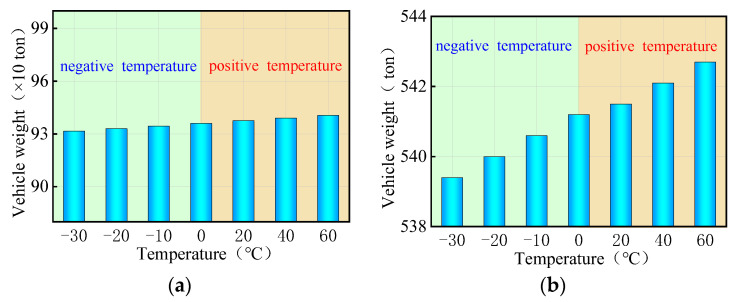
Ultimate vehicle weights of the overall overturning of the straight bridges subjected to trucks and uniform temperatures: (**a**) 3 × 25 m bridge and (**b**) 5 × 25 m bridge.

As can be seen in Figure 14, the ultimate vehicle weights for the straight bridges are affected differently by uniform temperature compared to curved bridges. Specifically, for the 3 × 25 m and 5 × 25 m straight bridges, the ultimate vehicle weights gradually increase with rising positive uniform temperatures, while they decrease with decreasing negative uniform temperatures. The ultimate vehicle weights of these straight bridges showed a slight increase of 0.1% to 0.5% with positive uniform temperatures between 20 °C and 60 °C, and a decrease of 0.1% to 0.5% with negative uniform temperatures between −10 °C and −30 °C.

In summary, it can be seen that the uniform temperatures, whether positive or negative, indirectly increase or decrease the ultimate vehicle weights of the bridge for overall overturning. The variations observed are consistently less than 1% and can be considered negligible. Consequently, uniform temperature has almost no effect on the anti-overturning stability of both curved and straight bridges. During the design and maintenance stages of bridges, it is not necessary to consider the effect of extreme overall temperature changes on the overall overturning stability of the bridge. This simplifies the design process and improves efficiency.

### 3.3. Gradient Temperature Effects

**Figure 15:**
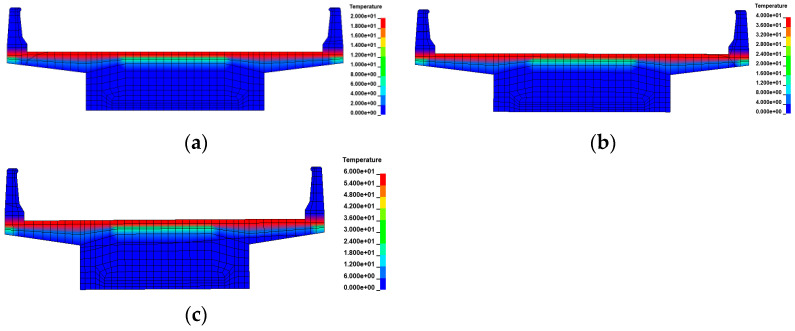
Thermodynamic FE models of the bridges under positive gradient temperatures: (**a**) heating 20 °C; (**b**) heating 40 °C; and (**c**) heating 60 °C.

**Figure 16:**
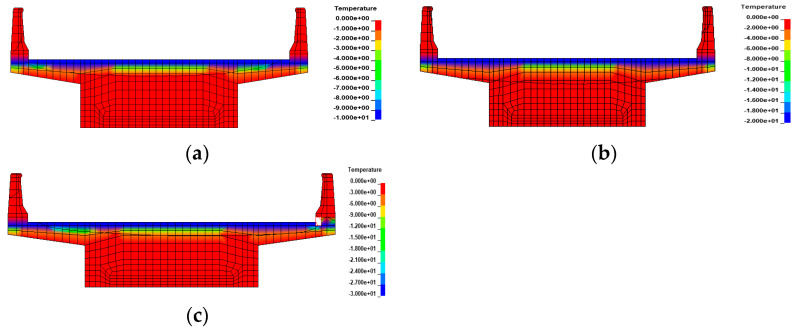
Thermodynamic FE models of the bridges under negative gradient temperatures: (**a**) cooling 10 °C; (**b**) cooling 20 °C; and (**c**) cooling 30 °C.

Numerical methods continued to be used to explore the effect of gradient temperatures on bridge overturning. In ANSYS/LS-DYNA, a gradient temperature was applied to nodes along the height of the girder. This involved using a gradient temperature distribution model (Figure 9) and recursive temperatures based on Equation (9). Two types of continuous box girder bridges, one with 3 × 25 m dimensions and the other with 5 × 25 m dimensions, were selected. These bridges have single-column piers and were characterized by radius curvatures of 80 m and 10,000 m to represent curved and straight bridges, respectively. The details of these bridges are consistent with those described in Section 2.1, and the modeling process aligns with Section 2.2. Various gradient temperatures, including positive conditions of 20 °C, 40 °C, and 60 °C, as well as negative conditions of −10 °C, −20 °C, and −30 °C, were considered to cover a range of extreme temperature scenarios. The thermodynamic FE models of a 3 × 25 m bridge under gradient temperature conditions are shown in Figure 15 and Figure 16.

**Figure 17:**
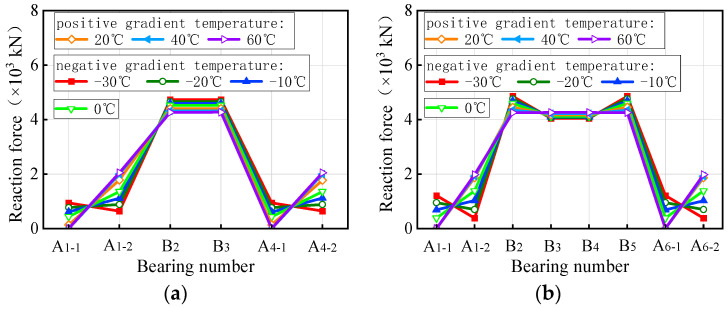
Bearing reactions of curved bridges subjected to gradient temperatures: (**a**) 3 × 25 m bridges and (**b**) 5 × 25 m bridges.

**Figure 18:**
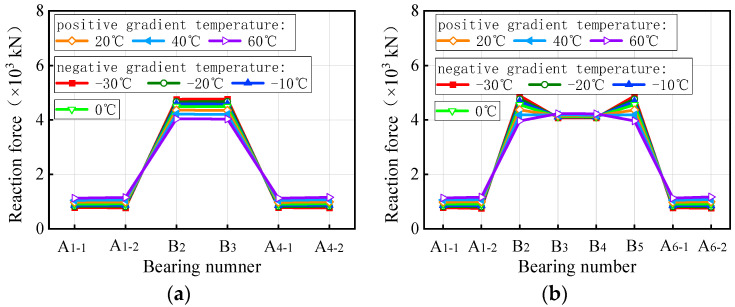
Bearing reactions of the straight bridges subjected to gradient temperatures: (**a**) 3 × 25 m bridge and (**b**) 5 × 25 m bridge.

The bearing reaction force of curved bridges under self-weight and a gradient temperature is depicted in Figure 17, while Figure 18 displays the results for straight bridges. In Figure 17, under positive gradient temperature conditions, the vertical reaction force of bearings A1-1 and A4-1 of the 3 × 25 m curved bridge gradually decreases with increasing temperature, reaching zero at a gradient temperature condition of heating 40 °C. Conversely, the vertical reaction force of bearing A1-2 shows an opposite trend, increasing by 51.0% at a temperature condition of heating 60 °C. Bearings B2 and B3 exhibit a similar pattern to A1-1, with a decrease of 5.7% at a temperature condition of heating 60 °C. For the 5 × 25 m curved bridge, the vertical reaction force of bearings (A1-1, A6-1) on the outside of the curvature decreases with temperature, while that of bearings (A1-2, A6-2) on the inside increases. The vertical reaction force of bearings B2 and B5 on the mid-pier decreases with temperature, while that of B3 and B4 increases. The vertical reaction force of the bearings on the 5 × 25 m curved bridge varies between −100.0% and 43.3% at a temperature condition of heating 60 °C. For the 3 × 25 m and 5 × 25 m curved bridges, the vertical reaction force under negative gradient temperatures exhibits an opposite pattern compared to positive gradient temperatures. When the bridges are subjected to a gradient temperature of cooling 30 °C, the vertical reaction force ranges from −52.8% to 117.6% and −72.5% to 213.2% for the two bridges, respectively. Notably, bearings A1-1 and A4-1 of the 3 × 25 m curved bridge failed at a gradient temperature of heating 40 °C, whereas bearings A1-1 and A6-1 of the 5 × 25 m curved bridge failed at a gradient temperature of heating 20 °C.

In Figure 18, the vertical reaction force of bearings A1-1, A1-2, A4-1, and A4-2 on the 3 × 25 m straight bridge increased gradually under positive gradient temperature conditions, reaching a maximum variation of 26.0% at a gradient temperature condition of heating 60 °C. Conversely, bearings B2 and P3 exhibited a contrary trend, with a maximum increase of 10.2% under the same heating conditions. Under negative gradient temperatures, the vertical reaction force of the bearings showed a reverse variation pattern compared to positive gradient conditions, ranging from −15.8% to 6.1% at a gradient temperature condition of cooling 30 °C for the 3 × 25 m bridge. Similarly, for a 5 × 25 m straight bridge, the vertical reaction force of all bearings varied from −17.1% to 7.2% under a gradient temperature range of −30 °C to 60 °C. Notably, the sum of the vertical reaction force of all bearings of the same bridge remained constant and unaffected by gradient temperatures. Overall, gradient temperatures only lead to a redistribution of vertical reaction force in both straight and curved bridges, without introducing additional force to the structural system.

**Figure 19:**
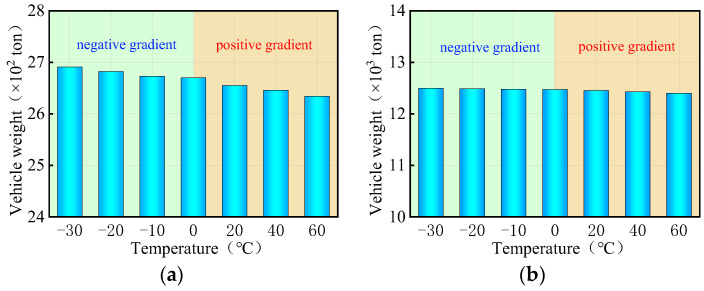
Ultimate vehicle weights of the overall overturning of the curved bridges subjected to gradient temperatures: (**a**) 3 × 25 m bridge and (**b**) 5 × 25 m bridge.

Figure 19 presents the ultimate vehicle weights for the 3 × 25 m and 5 × 25 m curved bridges subjected to three trucks and gradient temperatures. When exposed to positive gradient temperatures, the ultimate vehicle weights for both curved bridges decrease as the temperature rises, with a reduction of 1.4% observed at a gradient temperature of heating 60 °C. Conversely, under negative gradient temperatures, the ultimate vehicle weights gradually increase as the temperature drops. In the gradient temperature range of −10 °C to −30 °C, the ultimate vehicle weights of the curved bridges increase by 0.1% to 0.8%.

**Figure 20:**
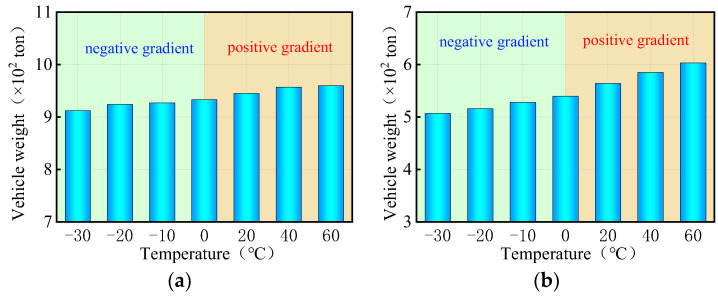
Ultimate vehicle weights of the overall overturning of the straight bridges subjected to gradient temperatures: (**a**) 3 × 25 m bridge and (**b**) 5 × 25 m bridge.

Figure 20 presents the ultimate vehicle weights for the straight bridges of 3 × 25 m and 5 × 25 m subjected to three trucks and gradient temperatures. Interestingly, the trend observed in the ultimate vehicle weights for straight bridges differs from that of curved bridges. Specifically, the ultimate vehicle weights for the 3 × 25 m and 5 × 25 m straight bridges increase with rising positive gradient temperatures, while they decrease with decreasing negative gradient temperatures. The ultimate vehicle weights of these straight bridges show an increase of 1.3% to 11.7% with positive gradient temperatures ranging from 20 °C to 60 °C, and a decrease of 0.6% to 6.1% with negative gradient temperatures ranging from −10 °C to −30 °C.

In all, the gradient temperature has inverse effects on the anti-overturning stability of curved bridges and straight bridges. The effect of gradient temperatures on the ultimate vehicle weights of the curved bridges is minimal, at less than 2%, and can be considered negligible. In contrast, the overturning effect of gradient temperatures is more pronounced on straight bridges, with a maximum effect of 11.7%. Therefore, it is essential to consider the adverse effects of gradient temperatures on the overturning stability of straight bridges in the design and construction process. Special attention must be paid to the risk of bridge overturning caused by temperature gradients during the design and construction of straight bridges.

## 4. Overturning Mechanism of Bridges Induced by Gradient Temperatures

To elucidate the overturning mechanism of curved and straight bridges under varying gradient temperatures, two thermodynamic coupled models were employed. The first model accounts for the coupling of bridge self-weight and a gradient temperature to analyze their effect on the bridge’s initial morphology. The second model considers the coupling of bridge self-weight, vehicles with ultimate weight, and a gradient temperature to assess the behavioral changes of the girder (displacement, rotation angle, and bearing reaction force) during the bridge’s overall overturning caused by gradient temperature fluctuations. In this model, the truck weight was set at the maximum value of the ultimate vehicle weights within the temperature range of −30 °C to 60 °C, as is seen in Figure 19 or Figure 20.

### 4.1. Overturning Mechanism for Curved Bridges

**Figure 21:**
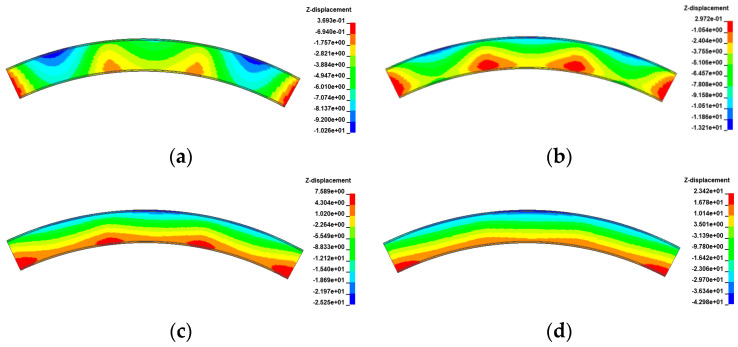
Vertical displacement of the 3 × 25 m curved bridge subjected to self-weight and positive gradient temperatures (unit: mm): (**a**) heating 0 °C; (**b**) heating 20 °C; (**c**) heating 40 °C; and (**d**) heating 60 °C.

**Figure 22:**
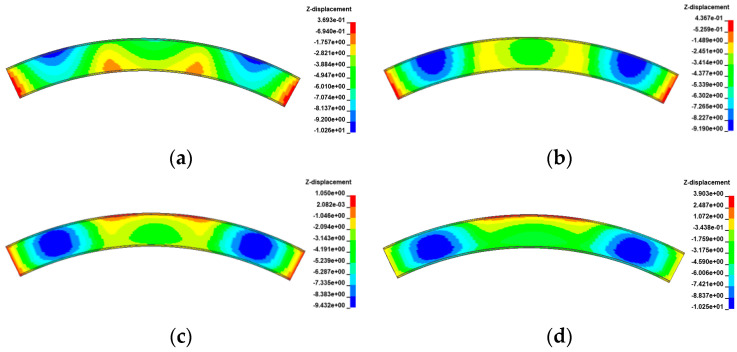
Vertical displacement of the 3 × 25 m curved bridge subjected to self-weight and negative gradient temperatures (unit: mm): (**a**) cooling 0 °C; (**b**) cooling 10 °C; (**c**) cooling 20 °C; and (**d**) cooling 30 °C.

**Figure 23:**
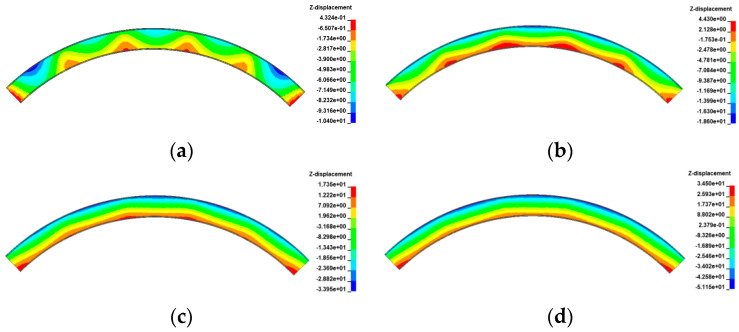
Vertical displacement of the 5 × 25 m curved bridge subjected to self-weight and positive gradient temperatures (unit: mm): (**a**) heating 0 °C; (**b**) heating 20 °C; (**c**) heating 40 °C; and (**d**) heating 60 °C.

**Figure 24:**
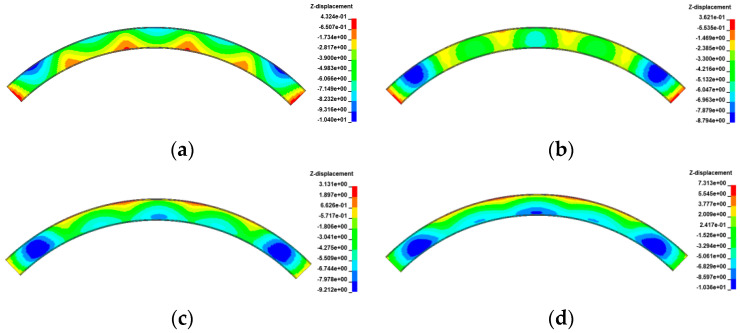
Vertical displacement of the 5 × 25 m curved bridge subjected to self-weight and positive gradient temperatures (unit: mm): (**a**) cooling 0 °C; (**b**) cooling 10 °C; (**c**) cooling 20 °C; and (**d**) cooling 30 °C.

Figure 21, Figure 22, Figure 23 and Figure 24 display the vertical deformation cloud diagrams of girders of the 3 × 25 m and 5 × 25 m curved bridges when subjected to the coupling effects of self-weight and a gradient temperature, respectively. Temperature variations have a notable effect on the vertical deformation of girders, causing opposite deformations on the inner and outer sides of the girder axis as the temperature fluctuates. The deformation of the girder mid-span section is particularly noticeable compared to the mid-pier girder section, transitioning from ‘down warp’ to ‘torsion’. When exposed to positive gradient temperatures, the girder torques outward towards the horizontal curvature of the girder axis, while under negative gradient temperatures, it torques inward.

**Figure 25:**
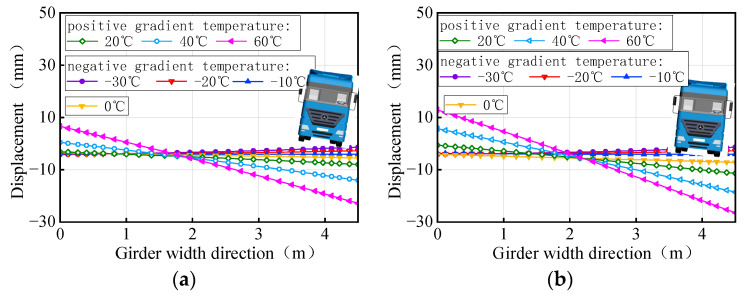
Vertical displacement of the mid-span section of the 3 × 25 m curved girder subjected to self-weight and gradient temperatures: (**a**) 3 × 25 m curved girder and (**b**) 5 × 25 m curved girder.

The vertical displacement of the mid-span section of the girder of the 3 × 25 m and 5 × 25 m curved bridges affected by the gradient temperatures is further plotted in Figure 25. This figure visually demonstrates the transition of the girder from ‘down warp’ to ‘torsion’. Notably, the vertical displacement changes are minimal near the support point or mid-span section along the bridge axis. Under the positive gradient temperatures, the torsional deformation towards the outer side of the horizontal curve, combined with the asymmetric truck loading, causes a shift in the center of gravity of the vehicle load towards the side prone to overturning, exacerbating the bridge’s instability. Conversely, torsional deformations induced by negative gradient temperatures occur toward the inner side of the horizontal curve and postpone the overturning of the bridge.

**Figure 26:**
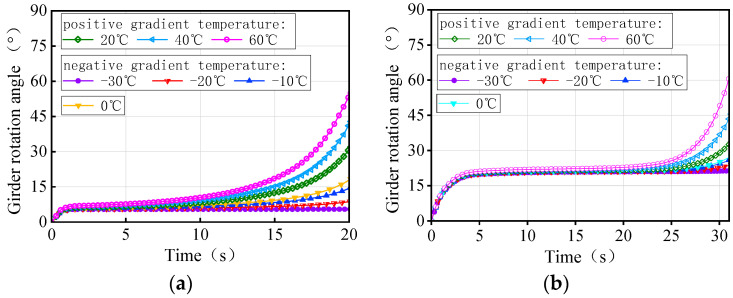
Rotation angle of the curved bridges subjected to self-weight, trucks, and gradient temperatures: (**a**) 3 × 25 m curved bridge and (**b**) 5 × 25 m curved bridge.

Figure 26 depicts the time history of the rotation angle for both 3 × 25 m and 5 × 25 m bridges under the same trucks and gradient temperatures, respectively. The maximum ultimate weight in Figure 19 is taken as the total weight for the three trucks in numerical simulations. There is a significant variation in the rotation angle of the girder with gradient temperature at the same moment, which is attributed to the difference in the initial torsional deformation of the girder. For the same vehicle load, the temperature conditions of the girder rotation angle from small to large at the same moment are −30 °C, −20 °C, −10 °C, 0 °C, 20 °C, 40 °C, and 60 °C. By analyzing Figure 19 and Figure 26 together, it becomes evident that a smaller rotation angle of the girder corresponds to a larger ultimate vehicle weight at the same moment, resulting in a greater anti-overturning stability of the bridge. Therefore, the rotation angle of a girder can serve as a crucial indicator of the level of the anti-overturning stability of a curved bridge.

**Figure 27:**
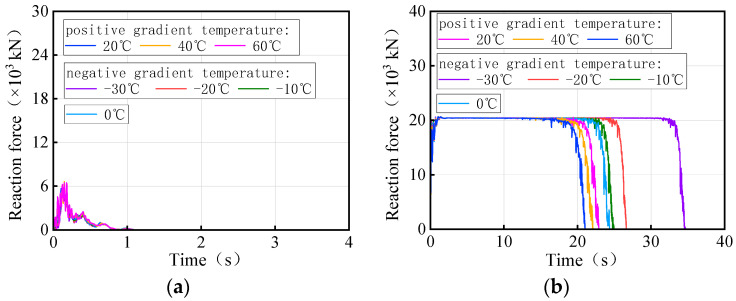
Vertical reaction force of the 3 × 25 m curved bridge subjected to trucks and gradient temperatures: (**a**) reaction force of bearing A1-2 and (**b**) reaction force of bearing B2.

**Figure 28:**
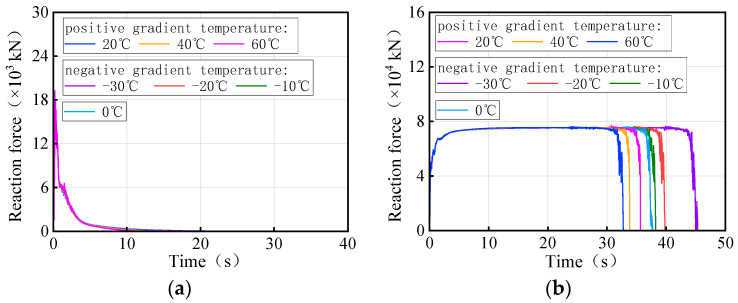
Vertical reaction force of the 5 × 25 m curved bridge subjected to trucks and gradient temperatures: (**a**) reactions of bearing A1-2 and (**b**) reactions of bearing B2.

Figure 27 and Figure 28 show the time history of the bearing reaction force for the 3 × 25 m and 5 × 25 m curved bridges under the same trucks and gradient temperatures, respectively. Considering that both the structure and the loads are symmetrical, a side-pier bearing and a mid-pier bearing were chosen for observation. For bearing A1-2, it is not easy to distinguish between temperature-influenced bearing reactions over time for the same truckload. However, bearing B2 exhibited a distinct pattern with time under the influence of gradient temperatures. There is a clear time sequence of gradient temperature-induced failures for these types of bearings, with higher temperatures more likely to cause bearing disengagement. For instance, in the case of 3 × 25 m and 5 × 25 m curved bridges under the same truckload, the sequence of bearing B2 disengagement time is observed to be 60 °C, 40 °C, 20 °C, 0 °C, −10 °C, −20 °C, and −30 °C. It is noted that for the same vehicle load, the later the mid-pier bearing failed, the higher the overall overturning capacity of the bridge. The effective service time of the mid-pier bearing is found to reflect the overall anti-overturning ability of the bridge. The mid-pier rotation angle is proposed as another evaluation index for assessing the level of anti-overturning stability of a curved bridge.

Overall, positive gradient temperature leads to the torsional deformation of curved bridges towards the outside of the curve, weakening anti-overturning stability. Conversely, negative gradient temperature causes torsional deformation towards the inside of the curve, delaying the overturning process. The smaller rotation angle of the girder with decreasing gradient temperature indicates higher anti-overturning stability. Additionally, the same bearing showed an obvious sequence of disengagement: the higher the gradient temperature, the earlier the bearing disengaged. The girder rotation angle and bearing reaction force are significant indicators for comparing the stability of curved bridges.

### 4.2. Overturning Mechanism for Straight Bridges

**Figure 29:**
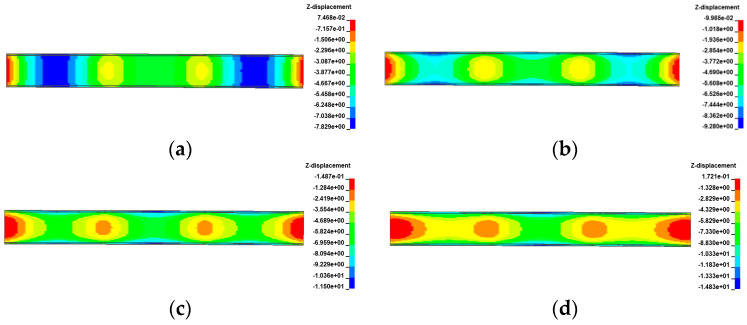
Vertical displacement of the 3 × 25 m straight bridge subjected to self-weight and positive gradient temperatures (unit: mm): (**a**) heating 0 °C; (**b**) heating 20 °C; (**c**) heating 40 °C; and (**d**) heating 60 °C.

**Figure 30:**
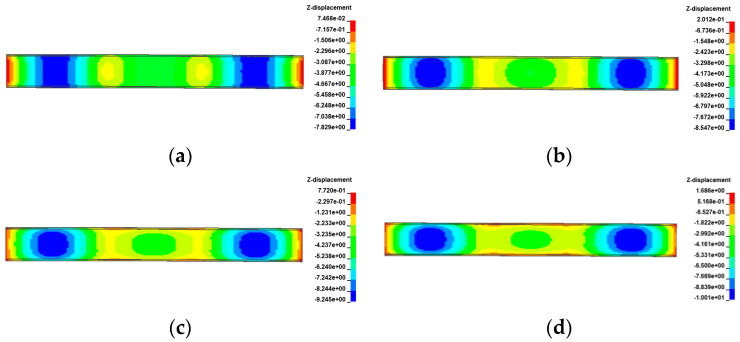
Vertical displacement of the 3 × 25 m straight bridge subjected to self-weight and negative gradient temperatures (unit: mm): (**a**) cooling 0 °C; (**b**) cooling 10 °C; (**c**) cooling 20 °C; and (**d**) cooling 30 °C.

**Figure 31:**
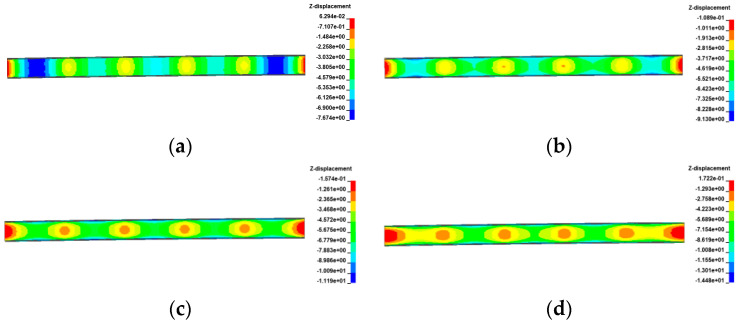
Vertical displacement of the 5 × 25 m straight bridge subjected to self-weight and positive gradient temperatures (unit: mm): (**a**) heating 0 °C; (**b**) heating 20 °C; (**c**) heating 40 °C; and (**d**) heating 60 °C.

**Figure 32:**
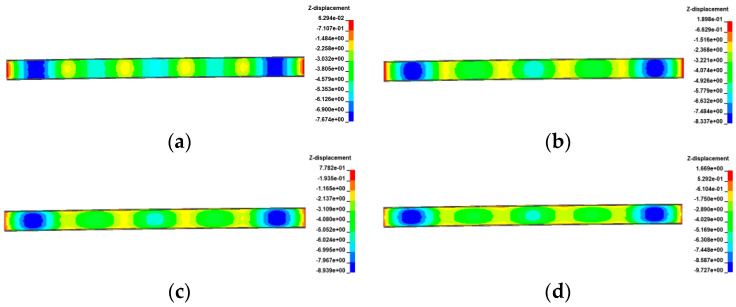
Vertical displacement of the 5 × 25 m straight bridge subjected to self-weight and positive gradient temperatures (unit: mm): (**a**) cooling 0 °C; (**b**) cooling 10 °C; (**c**) cooling 20 °C; and (**d**) cooling 30 °C.

Figure 29, Figure 30, Figure 31 and Figure 32 display the vertical deformation cloud diagrams of girders for both the 3 × 25 m and 5 × 25 m straight bridges, considering the coupling effects of self-weight and gradient temperatures. It is observed that temperature variations have a notable effect on the vertical deformation of girders, resulting in the overall ‘down warp’ and ‘upward warping’ of the girder as the temperature fluctuates.

**Figure 33:**
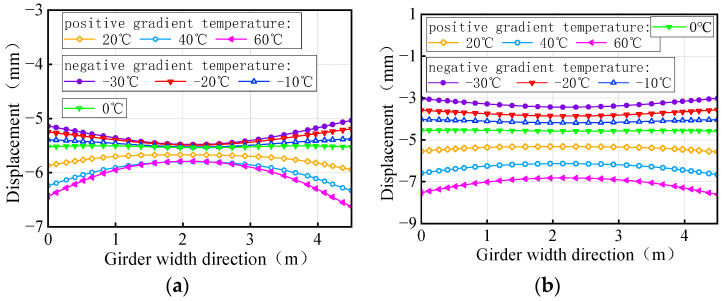
Vertical displacement of the mid-span section of the straight girder subjected to self-weight and gradient temperatures (unit: mm): (**a**) 3 × 25 m straight girder and (**b**) 5 × 25 m straight girder.

In comparison to the girder section at the mid-piers, the deformation of the girder mid-span section is clearly visible, as illustrated in Figure 33. As gradient temperature increases, this ‘down warp’ phenomenon prevents the separation of the girder from the mid-pier bearings. The four stages of the overturning process demonstrate that an effective mid-pier support can form a stable three-point support, crucial for maintaining the overall overturning stability of a straight bridge. Conversely, the upward warping resulting from a decrease in gradient temperature facilitates the separation of the girder from the mid-pier bearing.

**Figure 34:**
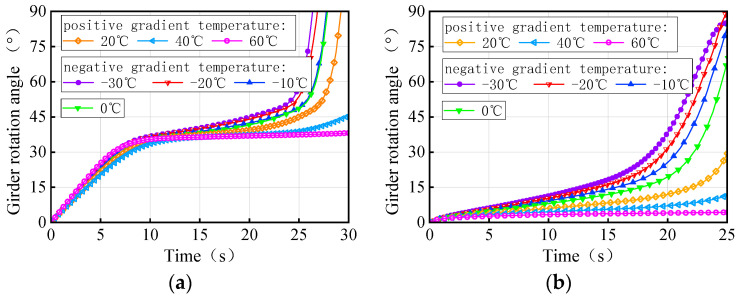
Rotation angle of the straight bridges subjected to trucks and gradient temperatures: (**a**) 3 × 25 m straight bridge and (**b**) 5 × 25 m straight bridge.

In the second type of thermodynamic model, the maximum ultimate weight in Figure 20 is taken as the total weight of the three trucks on the bridge. The time-history of the rotation angle for the 3 × 25 m and 5 × 25 m straight bridges is shown in Figure 34. Due to the initial deformation of the girder, there is a difference in the rotation angle of the girder subjected to different gradient temperatures at the same moment under the same trucks; the sequence of the rotation angle from small to large is 60 °C, 40 °C, 20 °C, 0 °C, −10 °C, −20 °C, and −30 °C. The relationship between the girder rotation angle and the ultimate vehicle weight, as demonstrated by Figure 20 and Figure 34, highlights that a smaller rotation angle results in a larger ultimate vehicle weight, and therefore the greater the anti-overturning performance of the bridge. The rotation angle of a girder can also be an important indicator of the level of anti-overturning stability of a straight bridge.

**Figure 35:**
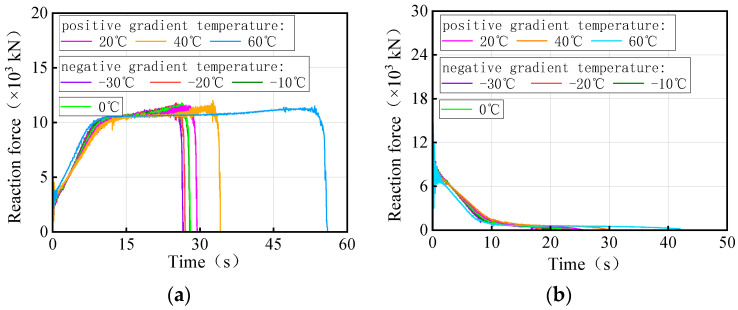
Vertical reaction force of the 3 × 25 m straight bridge subjected to trucks and gradient temperatures: (**a**) reaction force of bearing A1-2 and (**b**) reaction force of bearing B2.

**Figure 36:**
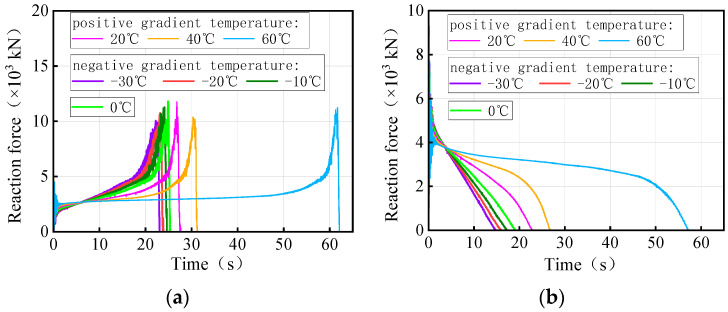
Vertical reaction force of the 5 × 25 m straight bridge subjected to trucks and gradient temperatures: (**a**) reaction force of bearing A1-2 and (**b**) reaction force of bearing B2.

Figure 35 and Figure 36 show the time-history of the bearing reaction force for the 3 × 25 m and 5 × 25 m straight bridges subjected to the same trucks and gradient temperatures, respectively. Bearings A1-2 (side-pier bearing) and B2 (mid-pier bearing) were chosen for observation. Both bearings exhibit distinct patterns over time in response to varying temperatures. There is an obvious time sequence of gradient temperature-induced failures of these types of bearings, with low temperatures being more likely to cause bearings to detach. For instance, the disengagement time sequence for the side-pier bearing and mid-pier bearing of 3 × 25 m and 5 × 25 m curved bridges, under the same truck loads, follows a pattern of −30 °C, −20 °C, −10 °C, 0 °C, 20 °C, 40 °C, and 60 °C. In the case of the same truckloads, a delayed failure of the bearings correlates with a higher overall overturning capacity of the bridge. The effective service time of the side-pier and mid-pier bearings can reflect the overall anti-overturning capability of the bridge. Therefore, the vertical reaction force of bearings can also serve as an evaluation index for assessing the level of anti-overturning stability in a straight bridge.

In summary, the increase in gradient temperature causes the straight girder to ‘down warp’, preventing it from separating from the bearing. Conversely, a decrease in gradient temperature promotes the separation process between the girder and the bearing. A higher gradient temperature results in a smaller girder rotation angle, indicating better anti-overturning performance. Lower temperatures lead to an earlier detachment of bearings, which is associated with higher bridge anti-overturning stability. For the same bearing, there was a noticeable time sequence of detachment associated with the gradient temperature. The higher the gradient temperature, the later the bearing failed. Girder rotation angle and bearing reaction force are significant indicators for comparing the stability of straight bridges.

The above studies focused on the overturning stability of box girder bridges under known temperature boundary conditions, excluding considerations of wind speed and solar radiation. Challenges remain in accurately simulating the complete process of bridge collapse within complex temperature fields, which encompass thermal radiation and thermal convection.

## 5. Conclusions

The study conducted a full-range nonlinear analysis of the lateral overturning process of a three-span curved continuous concrete box girder using the EFEM. The effects of uniform and gradient temperatures on the overall overturning stability of continuous curved and straight bridges were examined through numerical methods. The related mechanism by which temperature exacerbates or prevents bridge overall overturning was further investigated utilizing the temperature–bridge coupling thermodynamic model and temperature–vehicle–bridge coupling thermodynamic model. The conclusions drawn from this study are as follows:

(1) For the 3 × 25 m curved single-column pier bridge, the rotation angles of all girder sections are similar, with a maximum difference of 6°. During girder overturning, failed bearings initiate from the inner curvature or non-overturning side and progress towards the overturning side, leading to evolving support conditions for the bridge. The overall overturning collapse process of bridges can be divided into four stages: stabilization stage, transition stage, risk stage, and overturning stage. The state corresponding to the two-bearing support of the bridge can be used as a failure criterion to assess the limit state of the bridge’s overall overturning.
(2) As the uniform temperature fluctuates from −30 °C to 60 °C, the ultimate vehicle weights for the overall overturning of the 3 × 25 m and 5 × 25 m curved bridges gradually decrease by 0.1% to 0.7%. Conversely, the results for the straight bridges of the same dimensions showed an opposite trend, with an increase of 0.1% to 0.7% in ultimate vehicle weights. The effect of uniform temperatures on the ultimate vehicle weights of the bridges was found to be minimal, with changes of less than 1%, and can be considered negligible.
(3) The ultimate vehicle weights for the overall overturning of the 3 × 25 m and 5 × 25 m curved bridges decrease by 0.1% to 1.4% with a positive gradient temperature range of 0 °C to 60 °C. Conversely, under a negative gradient temperature range of 0 °C to −30 °C, the ultimate vehicle weights for the overall overturning of curved bridges increase by 0.1% to 0.8%. In contrast, the results for the 3 × 25 m and 5 × 25 m straight bridges show the opposite trend to the curved bridges. There is an increase in ultimate vehicle weights of 1.3% to 11.7% under positive temperature conditions and a decrease of 0.6% to 6.1% under negative gradient temperature conditions.
(4) The deformation of the girder of the curved bridges shifts from ‘down warp’ to ‘torsion’ as the gradient temperature changes. Positive gradient temperature leads to girder flips outward towards horizontal curvature, deteriorating the lateral stability of bridges, while inward flips improve stability. For the 3 × 25 m and 5 × 25 m curved bridges, bearing disengagement follows a specific temperature sequence. Delayed failures of mid-pier bearings at low temperatures are linked to higher overturning capacity, underscoring their significance in evaluating bridge stability.
(5) Gradient temperature variations can cause ‘down warp’ or ‘upward warping’ as a whole in straight girders, influencing their separation from mid-pier bearings. Within the range of −30 °C to 60 °C, the higher the temperature, the lower the girder rotation angle and the higher the anti-overturning stability. There is an obvious time sequence for bearing failure caused by temperature gradient: for the same bearing, the lower the temperature, the earlier the bearing will disengage. Both the girder rotation angle and reaction force of the bearing can serve as a comparative indicator of stability for straight bridges.
(6) This study focuses solely on the effect of temperature on the overall overturning mode of bridges. However, it is important to note that bridge failure modes also encompass girder slip failure, bearing extrusion failure, and pier failure. Investigating the influence and mechanisms of temperature on these specific damage modes may provide a more comprehensive understanding of bridge behavior. In addition, other factors affecting the stability of bridges are worth exploring, such as prestressing, foundation settlement, and some structural factors, and verifying by experimental trials.

## Data Availability

Data are contained within the article.
